# Molecular mechanism of SRP-dependent light-harvesting protein transport to the thylakoid membrane in plants

**DOI:** 10.1007/s11120-018-0544-6

**Published:** 2018-06-28

**Authors:** Dominik Ziehe, Beatrix Dünschede, Danja Schünemann

**Affiliations:** Molecular Biology of Plant Organelles, Ruhr-University Bochum, Universitätsstraße 150, 44780 Bochum, Germany

**Keywords:** LHCP, CpSRP, Transit complex, Alb3, CpFtsY, Thylakoid membrane

## Abstract

The light-harvesting chlorophyll a/b binding proteins (LHCP) belong to a large family of membrane proteins. They form the antenna complexes of photosystem I and II and function in light absorption and transfer of the excitation energy to the photosystems. As nuclear-encoded proteins, the LHCPs are imported into the chloroplast and further targeted to their final destination—the thylakoid membrane. Due to their hydrophobicity, the formation of the so-called ‘transit complex’ in the stroma is important to prevent their aggregation in this aqueous environment. The posttranslational LHCP targeting mechanism is well regulated through the interaction of various soluble and membrane-associated protein components and includes several steps: the binding of the LHCP to the heterodimeric cpSRP43/cpSRP54 complex to form the soluble transit complex; the docking of the transit complex to the SRP receptor cpFtsY and the Alb3 translocase at the membrane followed by the release and integration of the LHCP into the thylakoid membrane in a GTP-dependent manner. This review summarizes the molecular mechanisms and dynamics behind the posttranslational LHCP targeting to the thylakoid membrane of *Arabidopsis thaliana*.

## Introduction and overview of LHCP transport to the thylakoid membrane

**Fig. 1:**
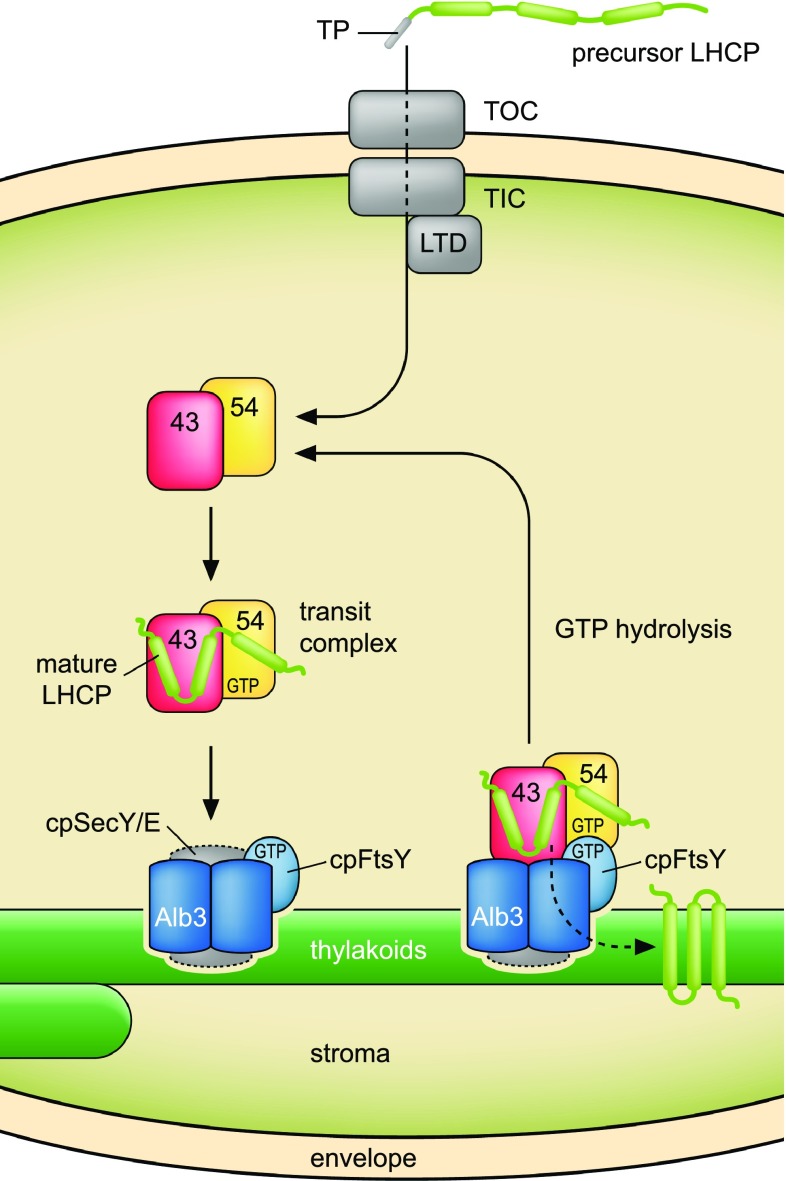
LHCPs are targeted to the thylakoid membrane via the posttranslational cpSRP-dependent transport pathway. LHCPs are imported posttranslationally into chloroplasts via the TOC/TIC translocon in the outer and inner envelope membrane. After import into the stroma, the transit peptide is cleaved off and the LHCPs are forwarded to the cpSRP complex by LTD. The transit complex consisting of cpSRP43, cpSRP54, and LHCP traverses the stroma and docks to the thylakoid membrane via interaction with cpFtsY and the Alb3 insertase. Alb3/cpFtsY are associated with the cpSecY translocase, which is, however, most likely not involved in the insertion process. GTP hydrolysis catalyzed by the SRP GTPases cpSRP54 and cpFtsY drives the dissociation of protein components

The capture of light energy is essential for biomass production through photosynthesis. In organisms ranging from green algae to vascular plants, photosystems I and II are associated with antenna complexes that consist of the light-harvesting chlorophyll a/b binding proteins (LHCPs) and are specialized for the harvesting and transfer of energy to the photosystems. LHCPs are integral thylakoid membrane proteins with three membrane-spanning regions and represent the most abundant proteins in this membrane system. LHCPs are encoded in the nucleus, translated in the cytosol, and targeted to the chloroplast via *N*-terminal transit sequences. Upon import into the chloroplast, which is mediated by two translocons in the outer and inner envelope membrane (TOC/TIC) (Jarvis 2008; Paila et al. 2015; Bölter and Soll 2016; Sjuts et al. 2017), the transit sequence is cleaved off (Richter and Lamppa 1999) (Fig. 1). The question of how the LHCPs are translocated through the stroma and subsequently inserted and assembled in the thylakoid membrane has been a subject of study for approximately three decades. In early studies, it was shown that a proteinaceous stromal factor is required for the formation of a soluble, stable ~ 120 kDa LHCP intermediate termed the transit complex, which traverses the stroma before thylakoid insertion (Fulsom and Cline 1988; Cline et al. 1989; Reed et al. 1990; Payan and Cline 1991). This factor was later identified as the so-called chloroplast signal recognition particle (cpSRP), which is located in the stromal fraction of the chloroplast (Li et al. 1995; Schünemann et al. 1998; Klimyuk et al. 1999). The cpSRP complex of higher plants is well characterized; it consists of two subunits, the conserved 54 kDa GTPase cpSRP54 and a unique chloroplast-specific 43 kDa protein, cpSRP43 (Franklin and Hoffman 1993; Schünemann et al. 1998; Klimyuk et al. 1999) (Fig. 1). CpSRP54 is homologous to cytosolic eukaryotic SRP54 and to the prokaryotic 54 homolog (Ffh) (Franklin and Hoffman 1993; Li et al. 1995), which are required for cotranslational protein transport to the endoplasmic reticulum and the plasma membrane, respectively (Akopian et al. 2013; Saraogi and Shan 2014; Voorhees and Hegde 2016). Consistent with the previous finding of a soluble LHCP intermediate, it has been demonstrated that complex formation between cpSRP and LHCP prevents aggregation of the hydrophobic LHCP in the aqueous milieu of the stroma and maintains it in an insertion-competent stage (Schünemann et al. 1998; Yuan et al. 2002; Goforth et al. 2004). The handover of the LHCP from the TOC/TIC import translocon to the cpSRP complex involves the ankyrin-repeat protein LTD (LHCP translocation defect), which is able to interact with the Tic machinery, LHCP, and cpSRP (Ouyang et al. 2011) (Fig. 1). Although cpSRP is sufficient to keep LHCP soluble and in an insertion-competent stage, the insertion of LHCPs into the thylakoid membrane requires additional factors. They comprise (i) the thylakoid membrane-associated SRP receptor cpFtsY (Kogata et al. 1999; Tu et al. 1999; Yuan et al. 2002), which is a homolog of the eukaryotic SRP receptor SRα and the prokaryotic FtsY, (ii) GTP, which is hydrolyzed by the SRP GTPases cpSRP54 and cpFtsY (Akopian et al. 2013) and (iii) the integral thylakoid membrane translocase Alb3 (albino 3) (Moore et al. 2000) (Fig. 1). Alb3 is a homolog of the bacterial YidC and mitochondrial Oxa proteins, which mediate the insertion, assembly, and folding of membrane proteins in the plasma membrane and inner mitochondrial membrane, respectively (Dünschede and Schünemann 2011; Wang and Dalbey 2011; Saller et al. 2012; Hennon et al. 2015).

In this review, we summarize the molecular details of the individual steps of posttranslational cpSRP-dependent LHCP transport in plants, including cpSRP43/cpSRP54 heterodimerization, cpSRP/LHCP transit complex formation, docking of the transit complex at the thylakoid membrane, and insertion of LHCP into the membrane. We also discuss aspects of the regulation and dynamics of the transport machinery. For information on the evolution of this transport system and on the overlapping function of cpSRP pathway components in the cotranslational transport of plastid-encoded proteins, we refer to previous reviews (Henry et al. 2007; Richter et al. 2010; Ziehe et al. 2017).

## Formation of the cpSRP43/54 heterodimer in *Arabidopsis thaliana*

**Fig. 2:**
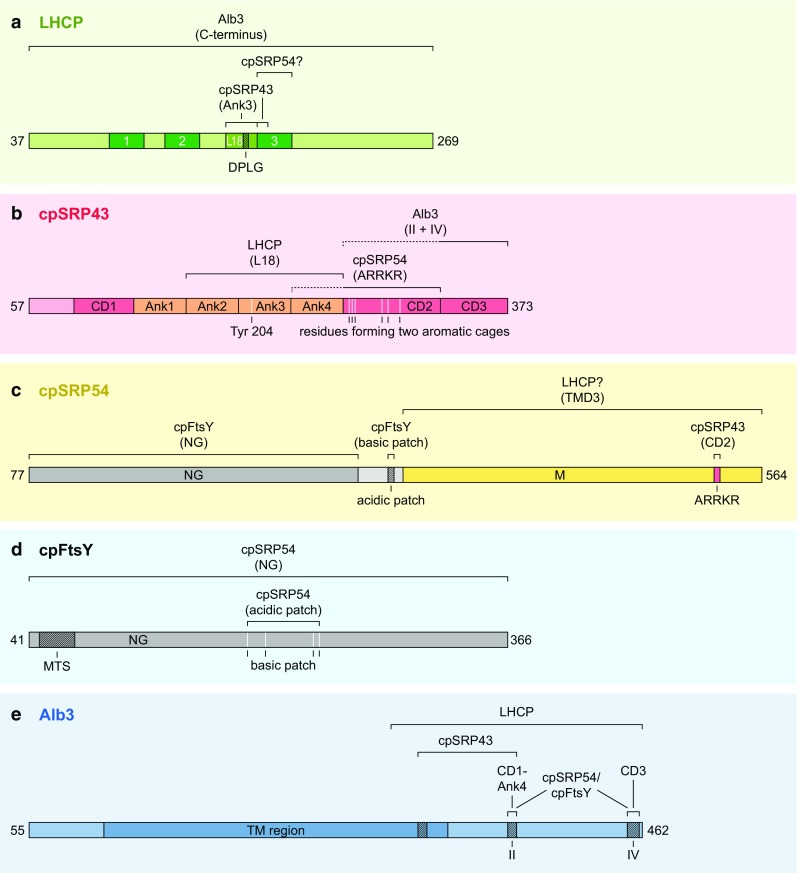
Scheme of protein–protein interactions within the posttranslational cpSRP transport pathway. The mature forms of the proteins involved in posttranslational LHCP transport and their functional domains are shown. Amino acid positions on the left and right correspond to the *Arabidopsis thaliana* proteins, except of LHCP, which refers to Lhcb1 of *Pisum sativum*. The diverse interaction partners and the interacting domains are indicated. Further details and references are given in the main text. **a** LHCP harbors three transmembrane domains (dark green, 1–3). The L18 region containing the crucial DPLG motif, which is responsible for cpSRP43 (Ank3) binding, is located between the second and third transmembrane domains. The binding region for cpSRP43 extends into LHCP’s transmembrane domain three. It is discussed if cpSRP54 binds to transmembrane domain three of LHCP. Furthermore, there is a direct interaction between Alb3’s *C*-terminus and LHCP. **b** CpSRP43 comprises three chromodomains (CD1-CD3, red) and four ankyrin repeats (Ank1-Ank4, orange). The Ank2-Ank4 region with the conserved Y204 binds the LHCP L18 peptide. The interaction with the ARRKR motif of cpSRP54 is accomplished via a twinned aromatic cage located in CD2, which is formed by six residues. A stimulating effect of Ank4 for cpSRP54/cpSRP43 complex formation was demonstrated (depicted by dashed line). CpSRP43 binds to the Alb3’s *C*-terminus (motifs II and IV) via CD2-3, whereby CD3 plays the major role (dashed and solid lines, respectively). **c** CpSRP54 is composed of a *N*-terminal NG domain (gray) and a *C*-terminal M domain (yellow) connected by a linker region (light gray). CpSRP54 binds to cpSRP43 with its *C*-terminal ARRKR motif (red) within the M domain. The NG domain binds to the homologous domain in cpFtsY. An acidic patch (dark shaded, E313; D314; E316; D317) next to the M domain forms an additional interaction site for cpFtsY. The M domain possibly also binds to the third transmembrane domain of LHCP. **d** Like cpSRP54, cpFtsY comprises a NG domain. A membrane targeting sequence (MTS, dark shaded) is located close to the *N*-terminus. As mentioned above cpFtsY interacts with cpSRP54 via its NG domain. Additionally, it contains a basic patch (K191; K193; K203; R204; K235; K236; K 240) as counterpart for cpSRP54’s acidic patch to provide an interaction via the complementary charged regions. **e** Alb3 is predicted to have five transmembrane domains which are summarized and depicted as transmembrane (TM) region (dark blue). CpSRP43 binds to a motif within the TM region, motif II and motif IV. Further data indicate that the binding to motif II and motif IV is mediated by CD1-Ank4 and CD3, respectively. The Alb3 *C*-terminus also binds the cpSRP54/cpFtsY complex, whereby the binding interface is probably provided by motifs II and IV. Additionally, LHCPs bind to the *C*-terminal region of Alb3

The chloroplast-specific cpSRP43 is a multidomain protein that consists of three chromodomains (CD1, CD2, CD3) and four ankyrin repeats (Ank1-Ank4) (Klimyuk et al. 1999; Goforth et al. 2004; Stengel et al. 2008). The *N*-terminal region of cpSRP43 harbors the first chromodomain (CD1), which is followed by 4 ankyrin repeats (Ank1-4) and two additional chromodomains (CD2, CD3) in the *C*-terminus (Fig. 2b). The second cpSRP subunit, cpSRP54, consists of an *N*-terminal *N* domain, a central G domain with GTPase activity and a methionine-rich M domain in the *C*-terminus (Franklin and Hoffman 1993) (Fig. 2c). In 2008, Stengel *et al*. published the first crystal structure of cpSRP43 (CD1-Ank4), revealing a unique arrangement of the chromodomains and the ankyrin repeats (Stengel et al. 2008) (Table 1). The crystal structure shows the characteristic helix-turn-helix motifs of Ank2 and Ank3 and the elongated nature of the Ank1 and Ank4 helices. CD1 is composed of three antiparallel β-sheets and a vertical α-helix that is oriented in the direction of the first ankyrin helix. Overall, the crystal structure reveals the elongated horseshoe character of the CD1-Ank4 region that is typical of ankyrin-repeat proteins.

**Table 1:** Crystal structures of proteins and protein complexes of the posttranslational cpSRP pathway

Macromolecule	Fragment	Organism	PDB number	References
cpSRP43				
cpSRP43 CD3	Residue: 319–368; His-tag cleaved off	*A. thaliana*	5E4X	Horn et al. (2015)
cpSRP43 CD1-Ank4	Residue: 85–267; His-tag cleaved off	*A. thaliana*	3DEO	Stengel et al. (2008)
cpSRP43/cpSRP54				
cpSRP43∆CD3 complexed with RRKRp	cpSRP43 residue: 85–318; His-tag cleaved off	*A. thaliana*	3UI2	Holdermann et al. (2012)
	cpSRP54 residue: 528–540; His-tag cleaved off	*A. thaliana*		
cpSRP43/LHCP				
cpSRP43 CD1-Ank4 complexed with L18	Residue: 85–267; His-tag cleaved off	*A. thaliana*	3DEP	Stengel et al. (2008)
cpSRP43/Alb3				
cpSRP43 CD2-CD3 fused via thioredoxin to Alb3 motif IV	Thioredoxin residue: 3-109; His-tag	*E. coli*	5E4W	Horn et al. (2015)
	cpSRP43 residue: 265–369	*A. thaliana*		
	Alb3 residue: 453–461; GST-tag cleaved off	*A. thaliana*		
cpFtsY				
cpFtsY	Residue: 80–383; His-tag	*P. patens*	4AK9	Träger et al. (2012)
cpFtsY	Residue: 24–112; thioredoxin	*A. thaliana*	2OG2	Chandrasekar et al. (2008)
cpFtsY	Residue: 65–366; His-tag cleaved off	*A. thaliana*	3B9Q	Stengel et al. (2007)
cpSRP54/cpFtsY				
cpSRP54 complexed with cpFtsY	cpSRP54 residue: 77–371; His-tag	*A. thaliana*	5L3R	Wild et al. (2016)
	cpFtsY residue: 80–366; His-tag	*A. thaliana*		

All available crystal structures and the corresponding PDB numbers are listed. Additional information about the crystallized fragments and the corresponding organism (*A. thaliana, Arabidopsis thaliana; P. patens, Physcomitrella patens; E. coli, Escherichia coli*) is given

CpSRP43/cpSRP54 complex formation was intensively studied by several groups. Initially, the cpSRP54 M domain was identified as the main binding region for cpSRP43 (Jonas-Straube et al. 2001; Groves et al. 2001; Goforth et al. 2004). Later, a 10-residue segment within the *C*-terminal tail region of cpSRP54M (RRKRp10) was shown to be important for cpSRP43 binding. This segment contains the conserved positively charged cpSRP43 binding motif ARRKR comprising residues 535–539 of cpSRP54 (Funke et al. 2005; Dünschede et al. 2015) (Fig. 2c). The formation of the cpSRP complex is mainly accomplished by the interaction of the ARRKR motif with cpSRP43-CD2. CD2 consists of three-stranded antiparallel β-sheets with a perpendicular α-helix and thus has the characteristic chromodomain architecture (Holdermann et al. 2012) (Table 1). In contrast to CD1, which is tightly connected to the ankyrin repeats, CD2 does not participate in any tertiary interactions with the *N*-terminal domains of cpSRP43 and is therefore more flexible. Within CD2 are located residues that form two aromatic cages that together present the binding interface for cpSRP54 (Holdermann et al. 2012) (Fig. 2b). Cage 1, which is formed by E268, W291 and D293, recognizes R537 of the cpSRP43 binding motif, whereas R536 is bound by the second aromatic cage, which consists of F267, Y269, and H304. Further detailed study revealed that the RRKRp10 peptide binds at the interface between CD2 and Ank4 and that in this complex CD2 is more closely positioned to Ank4 compared to free cpSRP43 (Holdermann et al. 2012). The importance of the Ank4 region for the cpSRP43/cpSRP54 interaction was also indicated by the observation that the affinity of binding of full-length cpSRP43 to RRKRp10 (*K*_d_ 0.39 µM) is significantly increased in comparison to that of cpSRP43 CD2 (Holdermann et al. 2012). Notably, full-length cpSRP54 and cpSRP54M bind cpSRP43 with even higher affinity (*K*_d_ 2–95 nM) (Hermkes et al. 2006; Gao et al. 2015; Ziehe et al. 2016) (Table 2), suggesting that additional regions of cpSRP54 are required to support high-affinity cpSRP complex formation. Little is known about the dynamics of the formation of this complex in vivo, but current data indicate that most if not all of the cpSRP43 is complexed with cpSRP54 in the stroma (Schünemann et al. 1998; Klimyuk et al. 1999).

**Table 2:** *K*_d_ values of protein–protein interactions within the posttranslational cpSRP-pathway of* Arabidopsis thaliana*

Interaction	Method	*K*_d_ [µM]	References
cpSRP43/Alb3			
43/Alb3 *C*-terminus	FA	12–18	Liang et al. (2016)
43/Alb3 *C*-terminus	ITC	5.1	Horn et al. (2015)
43/Alb3 *C*-terminus	ITC	9.7–13.2	Falk et al. (2010); Falk and Sinning (2010a, 2010b)
43/Alb3 *C*-terminus	ITC	0.094	Lewis et al. (2010)
cpSRP43/LHCP			
43/LHCP	LS	0.17/1.5	Liang et al. (2016)
43/LHCP + 54M		0.26	
43/LHCP	FA	0.14–0.3	Jaru-Ampornpan et al. (2010)
43/L18	ITC	0.322	Gao et al. (2015)
43/L18 + RRKRp10		0.107	
43/L18	ITC	1.17	Stengel et al. (2008)
43/L11	FA	0.022	Liang et al. (2016)
43/L11 + Alb3 *C*-terminus		0.011	
cpSRP43/cpSRP54			
43/54	MST	0.05	Ziehe et al. (2016)
43/54	FCS	0.095	Gao et al. (2015)
43/54M	SPR	0.0025	Hermkes et al. (2006)
cpSRP54/cpFtsY			
54/FtsY - PG	FRET	2.34	Chandrasekar and Shan (2017)
54/FtsY + PG		0.13	
54/FtsY*	FRET	0.77	Chandrasekar et al. (2017)
54NG/FtsY*		12	

Enlisted are the dissociation constants (*K*_d_ values) of protein complexes of the posttranslational cpSRP-pathway. Additionally, the methods by which the *K*_d_ values were determined as well as possible variations of the experimental settings are shown

*FA* fluorescence anisotropy,* FCS* fluorescence correlation spectroscopy,* FRET* forster resonance energy transfer,* ITC* isothermal titration calorimetry,* LA* light scattering,* MST* microscale thermophoresis, SPR surface plasmon resonance spectroscopy

* measured in the presence of 0.01 % Nikkol (mimicking of the effect of lipids)

## CpSRP binds LHCP to form a soluble LHCP transport intermediate, the transit complex

As described in the introduction, the LHC proteins are bound by the cpSRP complex in a way that maintains their solubility and insertion competence. Several studies have aimed to identify the intermolecular contacts between LHCP and the cpSRP subunits within the transit complex as summarized below.

Using various LHCP truncation constructs, an 18-residue-long binding site between the second and third transmembrane domains, L18 (VDPLYPGGSFDPLGLADD), and a hydrophobic region following the L18 motif were shown to be crucial for transit complex formation with cpSRP (DeLille et al. 2000) (Fig. 2a). The L18 motif harboring the DPLG sequence is conserved among LHCPs (Stengel et al. 2008; Barros and Kühlbrandt 2009) and therefore seems to be an important feature of members of this protein family. Tu et al. identified cpSRP43 as the binding partner for the L18 motif, while a direct interaction of cpSRP54 with LHCP was not detected (Tu et al. 2000). Further studies mapped the binding interface between LHCP and cpSRP via a pepscan analysis and confirmed the cpSRP43/L18 interaction (Groves et al. 2001). Cross-linking studies with pea Lhcb1 and cpSRP43 or a cpSRP complex revealed the presence of direct contacts between the L18 motif of Lhcb1 and the first part of TMD3 with cpSRP43; no contacts between Lhcb1 and cpSRP54 were detected (Cain et al. 2011). The structure of the cpSRP43/L18 complex was resolved by Stengel et al. (2008) (Table 1). CpSRP43 forms two predominantly hydrophobic grooves on its concave surface. L18 binds to groove 1, which is formed by ankyrin repeats 2–4. The DPLG motif is compactly folded and wraps around Y204 of Ank3 (Fig. 2b) and it was shown that mutations in the DPLG motif or in Y204 of cpSRP43 impair the cpSRP43/L18 interaction (Stengel et al. 2008). Studies to quantitatively analyze the interaction of cpSRP43 with the L18 region of LHCP reported dissociation constants (*K*_d_) ranging from 22 nM to 1.17 µM (Table 2). While the interaction between cpSRP43 and LHCP has been unambiguously proven, the question whether cpSRP54 contacts LHCP directly is less clear. As mentioned above, binding of cpSRP54 to LHCP was not observed in some studies, but other studies have reported evidence for cpSRP54/LHCP interaction. Initial reports revealed that cpSRP54 binds to residues within the third transmembrane domain (High et al. 1997; Groves et al. 2001) (Fig. 2c), emphasizing the importance of this transmembrane domain for transit complex formation (DeLille et al. 2000). Recently, it has been demonstrated that the absence of cpSRP54 or mutations within the M domain of cpSRP54 impair the formation of the cpSRP/LHCP transit complex (Dünschede et al. 2015; Henderson et al. 2016). Although the foregoing studies indicate that cpSRP54 plays an important role in transit complex formation, its precise contribution remains unclear. Apparently, it is not essential for the formation of soluble LHCP as it was shown that cpSRP43 alone acts as an ATP-independent chaperone for LHCP and is sufficient to maintain its solubility (Falk and Sinning 2010b; Jaru-Ampornpan et al. 2010). Therefore, cpSRP54 probably acts as an optimizing element that maintains the transit complex in an ideal insertion-competent state, thereby rendering the transport process more efficient (see also below in ‘Regulation and dynamics of the transport machinery’).

## Docking of the transit complex at the membrane and LHCP insertion

The cpSRP receptor cpFtsY binds peripherally to thylakoid membranes, and biochemical and genetic data prove that cpFtsY is linked to LHCP insertion (Kogata et al. 1999; Tu et al. 2000; Yuan et al. 2002; Tzvetkova-Chevolleau et al. 2007; Marty et al. 2009). Similar to cpSRP54, cpFtsY contains an NG domain that is necessary for GTP binding and hydrolysis (Fig. 2d). Crystal structures of various plant cpFtsY proteins reveal the characteristic four helix bundle within the N domain and the five G motifs within the G domain (Stengel et al. 2007; Chandrasekar et al. 2008; Träger et al. 2012) (Table 1). Tethering of cpFtsY to the membrane is mediated via an amphipathic helix located at the *N*-terminus (Stengel et al. 2007; Marty et al. 2009) (Fig. 2d). Within this region, two conserved phenylalanine residues, F48 and F49, are crucial for membrane binding, and it was demonstrated that cpFtsY is only functional in LHCP insertion when it is attached to the thylakoid membrane (Marty et al. 2009). CpFtsY is able to bind cpSRP54 and complex formation is established by interaction between the homologous NG domains of the two proteins (Jaru-Ampornpan et al. 2007; Stengel et al. 2007; Chandrasekar et al. 2008; Wild et al. 2016) (Table 1). Interestingly, recent data indicate that the M domain of cpSRP54 accelerates and stabilizes cpSRP54/cpFtsY complex formation via interaction between a positively charged cluster in the G domain of cpFtsY and a negatively charged cluster within the M domain of cpSRP54 (Jaru-Ampornpan et al. 2007; Chandrasekar et al. 2017) (Table 2). Furthermore, complex formation between cpFtsY and cpSRP54 is considerably stimulated by anionic phospholipids (Chandrasekar and Shan 2017) (Table 2).

In addition to cpFtsY, the integral thylakoid membrane protein Alb3 is involved in LHCP insertion. The characterization of Alb3 as the responsible insertase is based on the results of several studies that demonstrated specific inhibition of LHCP insertion by anti-Alb3 antibodies (Moore et al. 2000, 2003), and a direct interaction of Alb3 with components of the cpSRP transport pathway (Moore et al. 2003; Bals et al. 2010; Falk et al. 2010; Lewis et al. 2010; Dünschede et al. 2011; Walter et al. 2015; Chandrasekar and Shan 2017) (see also below). Consistently, the *alb3* null mutant in *Arabidopsis thaliana* displays an albino phenotype (Sundberg et al. 1997). Similar to bacterial YidC, the crystal structure of which was recently solved (Kumazaki et al. 2014a, b), Alb3 is predicted to contain five conserved transmembrane helices and a structurally disordered *C*-terminus protruding into the stroma of the chloroplast (Falk et al. 2010) (Fig. 2e). Blue native PAGE indicates that Alb3 can form dimers (Dünschede et al. 2011) (Fig. 1).

Several studies have described a direct interaction between Alb3 and cpSRP43 (Bals et al. 2010; Falk et al. 2010; Lewis et al. 2010; Dünschede et al. 2011; Liang et al. 2016). Two positively charged binding motifs within the *C*-terminus of Alb3, motif II (Dünschede et al. 2011; Falk et al. 2010) and motif IV (Falk et al. 2010), are important for cpSRP43 binding (Fig. 2e). Structural data revealed that motif IV binds to cpSRP43 CD3 (Horn et al. 2015) (Table 1). Biochemical data point to an interaction of motif II and cpSRP43 CD1-Ank4 (Liang et al. 2016). Furthermore, a binding site within the transmembrane region of Alb3 was described (Dünschede et al. 2011) (Fig. 2e). These data led to the conclusion that the transit complex is recruited to Alb3 via cpSRP43/Alb3 interaction. This docking model was further supported by the finding that cpSRP43 alone is able to keep LHCPs soluble (Falk and Sinning 2010b; Jaru-Ampornpan et al. 2010) and by the results of Tzvetkova-Chevolleau et al., who postulated an alternative LHCP transport pathway in *Arabidopsis thaliana* that bypasses cpFtsY and cpSRP54 but still requires cpSRP43 for LHCP targeting (Tzvetkova-Chevolleau et al. 2007). The latter authors demonstrated that the *ffc*/*cpftsy* double-knockout mutant lacking functional cpSRP54 and cpFtsY has a less severe phenotype and accumulates more LHCPs than the *cpftsy* single-knockout mutant. Therefore, these data provide support for an LHCP transport mechanism that depends on an efficient interaction between Alb3 and cpSRP43. However, the dissociation constant of the cpSRP43/Alb3 *C*-terminus interaction was described inconsistently. Whereas a *K*_d_ of ~ 90 nM, indicating high-affinity binding, was reported by Lewis et al. (2010), other reports point to a rather weak, transient interaction (*K*_d_ 5–18 µM) (Falk et al. 2010; Falk and Sinning 2010a; Horn et al. 2015; Liang et al. 2016) (Table 2). Notably, the affinity of cpSRP43 for full-length Alb3 has not been determined yet. Therefore, the contribution of the Alb3/cpSRP43 interaction to the recruitment of the transit complex to the membrane remains unclear.

Other data support the existence of an alternative LHCP targeting mode in which the transit complex recruitment to Alb3 is accomplished primarily via an interaction between Alb3 and the cpSRP54/cpFtsY complex. The studies of Moore et al (2003) indicate that Alb3 can bind the cpSRP54/cpFtsY complex even in the absence of cpSRP43 and LHCP, and a recent study reported that Alb3 *C*-terminus binds the cpSRP54/cpFtsY complex with an affinity in the submicromolar range (Chandrasekar and Shan 2017). Additional data suggested that motifs II and IV within Alb3 *C*-terminus, which are responsible for the cpSRP43 interaction, might also play a role in binding the cpSRP54/cpFtsY complex (Chandrasekar and Shan 2017) (Fig. 2e).

Various studies have demonstrated that LHCP insertion is Alb3-dependent and independent of the thylakoid membrane cpSecY/E translocase (Mori et al. 1999; Moore et al. 2003). However, a direct association between Alb3 and the cpSecY translocase has been shown by coimmunoprecipitation experiments, double immunogold labeling and cross-linking studies, while there is no clear evidence for the presence of an uncomplexed pool of Alb3 (Klostermann et al. 2002). The Alb3/cpSecY translocase association was confirmed by Moore et al. (2003) who showed in diverse precipitation analyses that a stabilized complex consisting of cpFtsY and cpSRP can precipitate Alb3 and cpSecY from solubilized thylakoid membranes. Interestingly, recent data obtained in comigration and coimmunoprecipitation analyses of solubilized thylakoid membrane complexes indicate that cpFtsY and Vipp1 are additional components of the Alb3/cpSecY-containing complex in the thylakoid membrane (Walter et al. 2015). Therefore, it seems possible that the transit complex docks to a preformed cpFtsY/Alb3/cpSecY complex in the thylakoid membrane; however, the cpSec translocase does not appear to be involved in contact formation or the insertion process (Fig. 1).

## Regulation and dynamics of the transport machinery

The nucleotide requirement for LHCP integration was examined by *in vitro* reconstitution assays in two main studies. Initially, Hoffman and Franklin (1994) showed that GTP is the only nucleotide required for integration and demonstrated an inhibitory effect of non-hydrolyzable analogs of GTP (Hoffman and Franklin 1994). The requirement for GTP hydrolysis in LHCP insertion was confirmed by Yuan et al. (2002). Notably, this study also described a stimulatory effect of ATP and of the non-hydrolyzable analog AMP-PNP, indicating that a yet unknown ATP-binding protein might be involved in LHCP integration. GTP is not required for formation of the transit complex (Yuan et al. 2002); however, it is important for triggering the GTPase cycle of the cpSRP54/cpFtsY complex at the membrane (Jaru-Ampornpan et al. 2007, 2009), which is a multistep process comprising assembly of the GTP-loaded cpSRP54/cpFtsY complex, reciprocal GTPase activation and dissociation of the complex. Interestingly, within the GTPase cycle, the cpSRP54/cpFtsY assembly step plays a crucial role in LHCP insertion, and GTPase activation enhances the insertion efficiency to some extent (Nguyen et al. 2011). Molecular dynamic simulations indicate that binding of GTP to cpFtsY is an important step in cpSRP54/cpFtsY complex formation because it induces conformational changes in cpFtsY that favor the formation of a complex with cpSRP54 (Yang et al. 2011), which is a kinetically fast interaction (Jaru-Ampornpan et al. 2007). In recent years, several mechanisms that regulate the GTPase activity of the individual SRP GTPases and of the cpSRP54/cpFtsY complex have been described. GTPase assays using the soluble recombinant cpSRP54/cpFtsY complex were used to demonstrate that cpSRP43 and the *C*-terminus of Alb3 stimulate GTP hydrolysis by the complex and that the stimulatory effect of Alb3 *C*-terminus is strictly coupled to the presence of cpSRP43 (Goforth et al. 2004; Lewis et al. 2010). A regulatory effect of Alb3 *C*-terminus on GTP hydrolysis by the cpSRP54/cpFtsY complex was also described by Chandrasekar and Shan (2017). However, in that case Alb3 *C*-terminus had an inhibitory, cpSRP43-independent effect on GTP hydrolysis, which led to the hypothesis that this negative regulation might enable positioning of the transit complex on the translocase and transfer of LHCP to Alb3 before GTP hydrolysis occurs. The inconsistent findings are probably due to the use of different experimental conditions. Chandrasekar and Shan (2017) performed GTPase assays in the presence of PG liposomes and reported that the regulatory effect of Alb3 *C*-terminus on GTPase activation is dependent on the presence of anionic phospholipids (Chandrasekar and Shan 2017). The role of lipids in regulating the GTPase cycle is further supported by the finding that liposomes stimulate the basal GTP hydrolysis rate of cpFtsY (Marty et al. 2009).

Although knowledge of the dynamics of the transport machinery is rather limited, some of the mechanisms (besides regulation of the GTPase cycle) involved in coordinating the order of events have recently been elucidated. A central role is assigned to cpSRP43 because it shows high interdomain dynamics, a feature that probably enables it to undergo flexible interactions with its several binding partners (Gao et al. 2015) (Fig. 2b). The binding of cpSRP54 to cpSRP43 reduces the flexibility of cpSRP43 (Gao et al. 2015) and induces a conformational change (Liang et al. 2016) that results in an enhanced binding affinity of cpSRP43 to LHCP (three to sixfold) (Gao et al. 2015; Liang et al. 2016). The affinity between the activated cpSRP43 and the L18 motif of LHCP was determined to be in the range of 100–300 nM (Gao et al. 2015; Liang et al. 2016) (Table 2). The release of LHCP from cpSRP is triggered upon interaction of cpSRP43 with the insertase Alb3, as it was shown that the addition of recombinant Alb3 *C*-terminus dissociates soluble cpSRP43/LHCP complexes (Lewis et al. 2010; Liang et al. 2016) and that this effect is coupled to the presence of the cpSRP43 binding motifs II and IV in Alb3 *C*-terminus (Liang et al. 2016). Furthermore, it was observed that Alb3 *C*-terminus weakens the interaction between cpSRP43 and the RRKR10p peptide, leading to the hypothesis that this might contribute to the release and transfer of LHCP to the insertase (Falk et al. 2010; Horn et al. 2015).

## The thylakoid membrane as the site of cpSRP-dependent LHCP insertion and pigment loading

Approximately 30 years ago, it was demonstrated in *in vitro* experiments that LHCP is inserted into thylakoid membranes but not into envelope membranes (Cline 1986). In later studies, the *in vitro* insertion assay of LHCP into thylakoids has been extended and successfully used by several groups to study the molecular details of this pathway (see above and Kuttkat et al. 1995). The thylakoid membrane as the site of cpSRP-dependent LHCP insertion is further supported by the exclusive localization of the Alb3 translocase in thylakoid membrane (Gerdes et al. 2006). *In vivo* data support the important role of cpSRP-dependent LHCP transport; the *ffc*/*chaos* double-knockout mutant, which lacks cpSRP54 and cpSRP43, showed pale green leaves due to the loss of 85% of its chlorophyll as well as a strong decrease in the levels of most LHCPs and greatly reduced number of thylakoids (Amin et al. 1999; Hutin et al. 2002). For a detailed summary of the phenotypes of cpSRP pathway mutants, we refer to previous review articles (Schünemann 2004; Henry et al. 2007; Richter et al. 2010). The biogenesis of stable LHC complexes in the thylakoid membrane requires assembly with pigments (Kuttkat et al. 1995, 1997; Plumley and Schmidt 1995; Tanaka and Tanaka 2011). Because the soluble LHCP/cpSRP transit complex forms in the absence of pigments and *in vitro* experiments have demonstrated that inserted LHCP is complexed with pigments and assembled in trimers (Kuttkat et al. 1995), it is very likely that pigment loading occurs at the site of insertion in the thylakoid membrane. This is consistent with the finding that the chlorophyll (chl) b-deficient *Arabidopsis thaliana cao*-1 mutant can efficiently import LHCPs, while stable assembly with PSII is affected (Nick et al. 2013). Furthermore, studies using a chl b-deficient *Chlamydomonas reinhardtii* mutant point to an interconnection between pigment synthesis and LHCP biogenesis that occurs at the thylakoid membrane (Plumley and Schmidt 1995). However, Reinbothe et al. (2006) observed severely impaired LHCP import into chloroplasts from a chl b-deficient mutant of *Arabidopsis thaliana* (Reinbothe et al. 2006), and studies with *Chlamydomonas reinhardtii* mutants showed that the absence of chl b led to an accumulation of LHCPs in the cytosol and the vacuole (Park and Hoober 1997). Based on that evidence, a model of LHCII assembly was hypothesized in which chl b is incorporated into LHCP in the envelope membrane during import. Non-pigment-loaded LHCP would reenter the cytosol for degradation (Hoober et al. 2007). The transfer of the pigment-loaded LHCII from the envelope to the thylakoid was hypothesized to be mediated by vesicles. Indeed, in recent years, there has been increasing evidence for the presence of a vesicle transport system in chloroplasts. However, the question of whether or not proteins are transported in addition to lipids has not yet clearly been answered (Karim et al. 2014; Karim and Aronsson 2014; Lindquist et al. 2016). Tanz et al. (2012) suggested a vesicle-based transport of LHCPs predominantly in cotyledons. As described above, the *ffc*/*chaos* mutant shows a severely compromised phenotype but still contains residual levels of LHCPs, indicating that at least some members of the LHCP family can be transported in a cpSRP43/54-independent manner in these plants. Considering that the upregulation of stromal chaperones such as ClpC plays a role in compensating for the absence of cpSRP54 in *ffc* mutants (Rutschow et al. 2008) and that yeast mutants compensate for the loss of the SRP-pathway by a reduced growth rate and induction of heat shock proteins (Mutka and Walter 2001), it is likely that the cpSRP double mutant uses similar strategies to adapt to the loss of cpSRP. Taken together, the model of vesicle-mediated transport of (pigment-loaded) LHCP remains speculative, at least for chloroplasts of higher plants, whereas the current *in vitro* and *in vivo* data indicate that cpSRP-dependent LHCP transport, which includes insertion and pigment loading at the thylakoid membrane, plays a primary role in LHCP biogenesis in higher plants.

## Conclusions

In the last decades, considerable progress has been made in understanding the molecular details of cpSRP/Alb3-dependent LHCP transport to the thylakoid membrane. However, several central issues need to be investigated in the future to get a more complete picture of LHCP transport. To further decipher this mechanism, it is important to get more structural information about single components and protein complexes. Here, it will be especially challenging to elucidate the structure of the transit complex, the Alb3 insertase, and finally the docking complex. In addition, little is known about the spatiotemporal coordination of LHCP transport, insertion, and pigment delivery/assembly.
